# Recalcitrant Septic Nonunion of the Ulna

**DOI:** 10.7759/cureus.7195

**Published:** 2020-03-06

**Authors:** Niels Bech, Peter Kloen

**Affiliations:** 1 Orthopedic Surgery, Amsterdam University Medical Center, Amsterdam, NLD

**Keywords:** nonunion, infection, ulna fracture

## Abstract

An infected diaphyseal forearm nonunion can be a challenge. After several failed salvage procedures, the patient can be left with residual pain, shortening, bone loss, and poor soft tissue envelope. Keystones for infected nonunion treatment are debridement, cultures, antibiotics, stability, and restore alignment. This report describes the current literature on the treatment of forearm nonunion, and we present a case of a recalcitrant infected ulna nonunion that ultimately healed after 12 surgeries.

## Introduction

Modern plate and screw fixation has solved the problems posed by diaphyseal forearm fractures since the late 1950s, with very high rates of uneventful healing (>95%) [1]. Nonunions do occur and are most often due to technical insufficiencies and/or infection.

The case presented here shows that despite a step-wise strategy using basic surgical principles, immediate success is not always a given. At each step, there are several surgical options and there is no clear evidence which option is better [2]. The final result of this recalcitrant case shows the inherent healing capacity of bone that will heal when mechanics and biology are optimized while providing an infection-free environment.

## Case presentation

**Figure 1:**
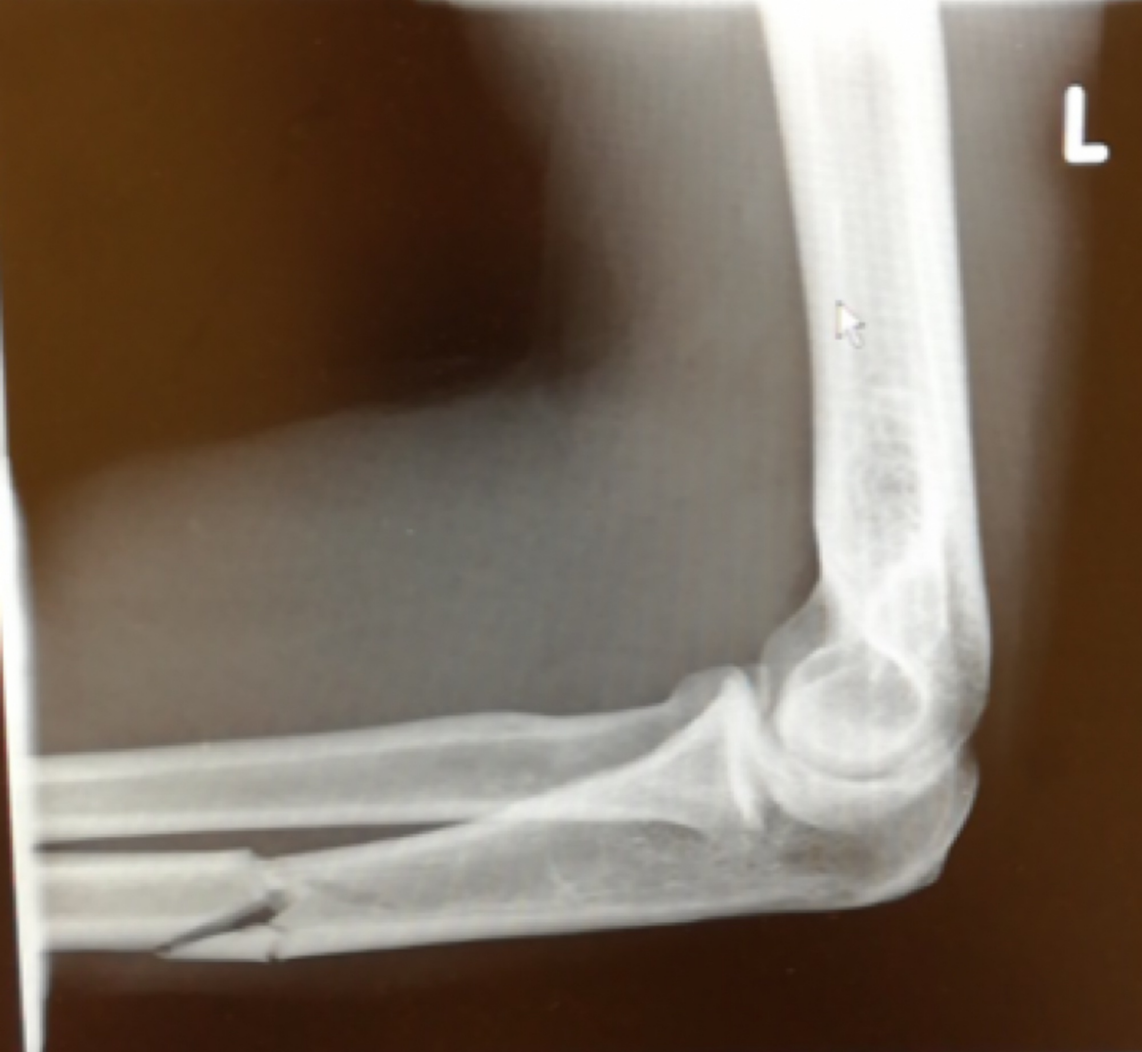
AO type 2U2B3 fracture of the left ulna

**Figure 2:**
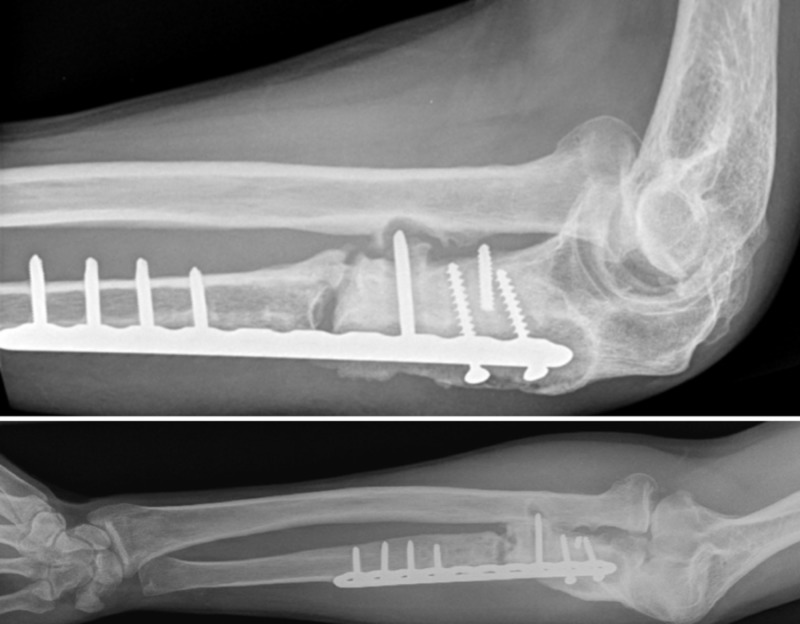
On first presentation at our clinic Note the ulna bone loss and the shortening and incongruency in the distal radio-ulnar joint

A 37-year-old, right-handed male was assaulted and sustained a mild closed head injury and a diaphyseal fracture of his proximal ulna AO/OTA type 2U2B3 (Figure 1). His past medical history was significant for COPD and he was a smoker. Primary ulna plating was done at an outside hospital. Early failure of fixation and infection led to six additional operations at the initial hospital over a five-year period. He was then referred to us with a stiff elbow, failed fixation on X-rays (Figure 2), and a septic nonunion with a pus-draining fistula.

There was an ulna minus deformity (Figure 2). Based on the radiographs and the draining fistula, the nonunion was classified according to Cierny as a type 4 septic nonunion (diffuse osteomyelitis) [3].

Our first surgery consisted of thorough debridement with the removal of all loose implants and dead bone. The fistula was excised. Six deep cultures were obtained, including five tissue cultures and one screw. After the cultures were taken, intravenous cefazolin (2 gram) was administered. All sclerotic, necrotic, or avascular bone was removed, as well as all inflammatory and fibrous intervening tissue. The bone marrow canal was opened with a 2 mm drill on both ends of the nonunion. The area was copiously irrigated with 9 liters NaCl using low flow irrigation.

**Figure 3:**
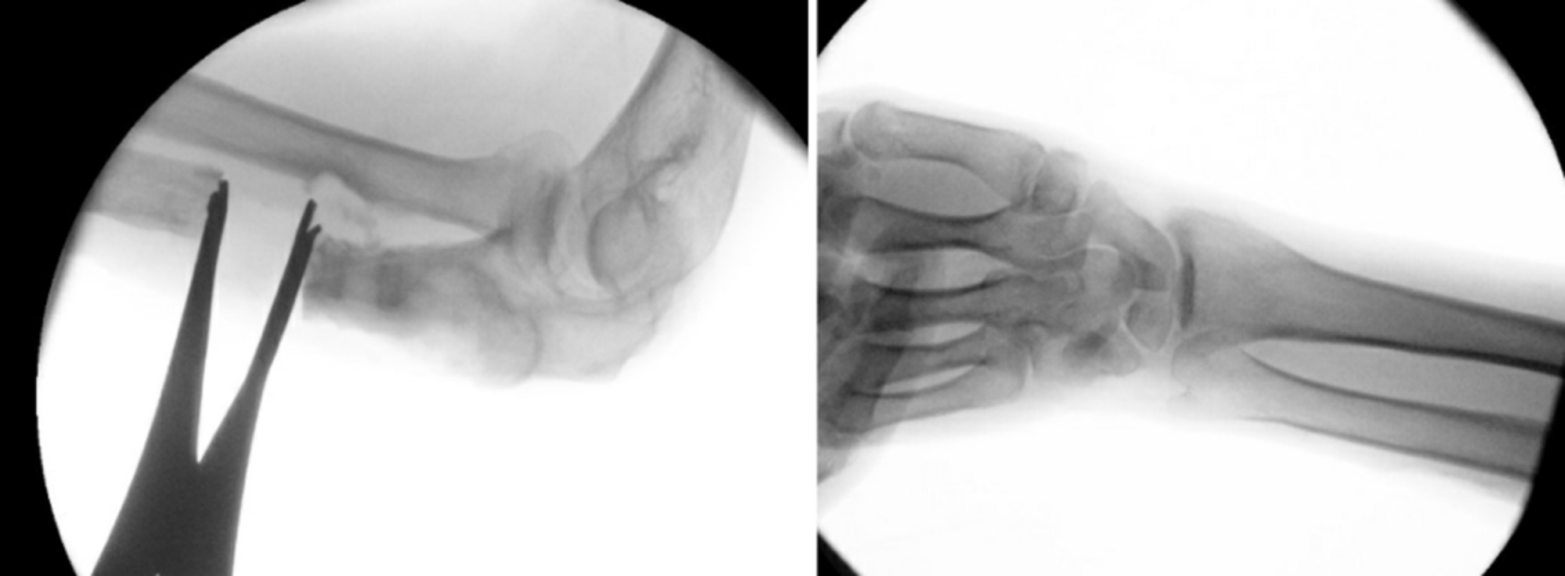
Perioperative imaging of lengthening of the ulna

**Figure 4:**
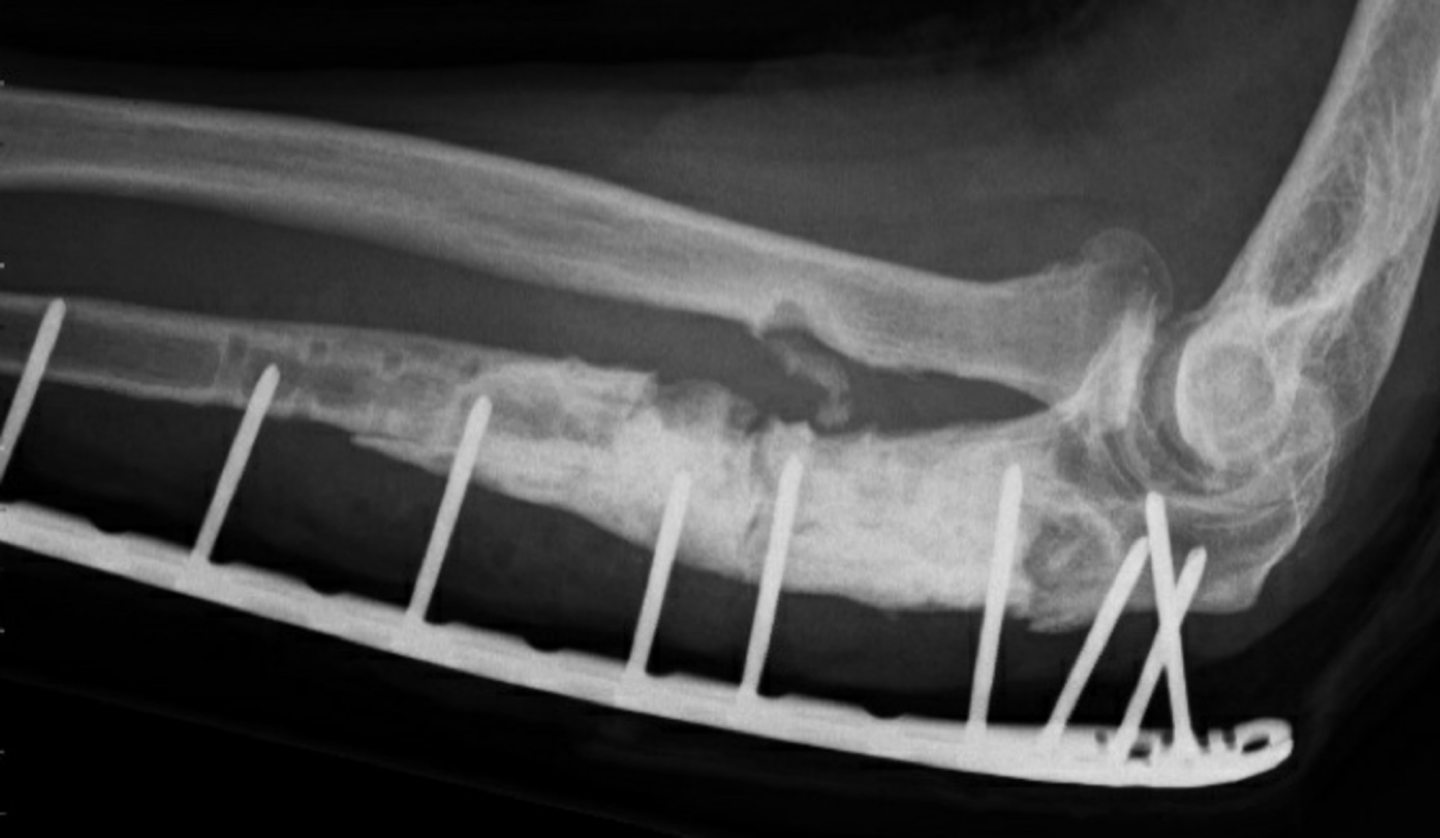
Result after Masquelet stage 1 A PMMA spacer and the plate is used as an external fixator PMMA: Polymethyl methacrylate

Using his contralateral healthy wrist as a template, we lengthened the ulna under fluoroscopic control to match the length of the uninvolved ulna (Figure 3). This resulted in a defect of 2.5 cm at the level of the nonunion (Figure 3). Polymethyl methacrylate (PMMA) cement with gentamycin and additional vancomycin powder (2 gram) was mixed and placed in the defect (Figure 4). Once the cement hardened, the excess cement was removed. Skin and underlying soft tissue were closed in a single layer with a vertical mattress stitch technique.

For stability, we applied a titanium proximal humerus plate (Philos plate, DePuy-Synthes, Amersfoort, The Netherlands) as an external fixation plate. Locking screws were placed via stab incisions in the skin (Figure 4). Postoperatively, the patient was allowed to use his arm freely, with restrictions to 5 kg weight-bearing. Five out of six cultures grew Staphylococcus epidermidis. After consultation with our hospital infectious disease specialist and a microbiologist, the final antibiotic treatment consisted of two weeks vancomycin intravenous (IV) followed by four weeks clindamycin orally.

**Figure 5:**
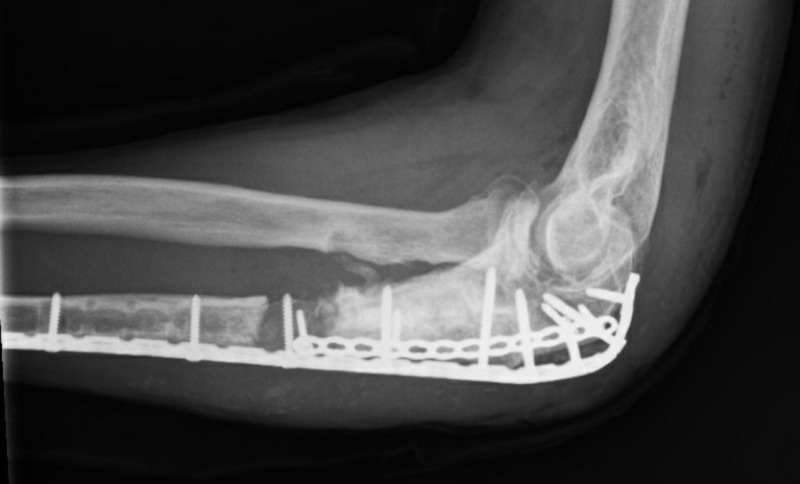
After Masquelet stage 2 Double plating and a tricortical iliac crest grafted in the bone defect

Wound healing was without complications. Six weeks after the first surgery, the patient was taken back to the operating room for the second stage of the Masquelet technique: removal of the plate, removal of the bone cement, and placement of a 2.5 cm intercalary iliac crest tricortical bone graft. Fixation was done with two plates (Figure 5). Again, five deep cultures were taken. After two weeks, the cultures were negative and no further antibiotic treatment was indicated. Of note is that he had not had an antibiotic holiday so these last cultures were taken under antibiotic regiment. Two weeks later, he, unfortunately, returned with pus draining from the wound. The wound was opened in the operating room, cultures were taken, and irrigation and debridement were done. All hardware seemed stable and solid; no hardware was removed.

Cultures grew Escherichia coli, Staphylococcus epidermidis, Enterococcus faecalis, and Streptococcus pneumonia. His antibiotic treatment consisted of two weeks of cefazolin IV followed by oral co-cotrimoxazole for 10 weeks.

**Figure 6:**
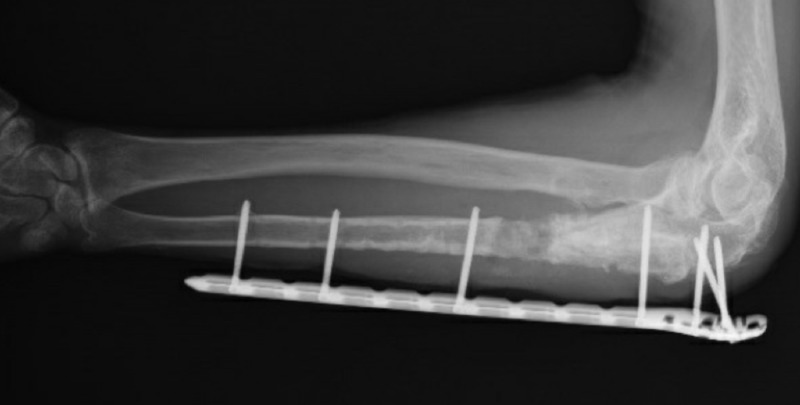
Both plates were removed because of infection and a new plate was placed as an external fixator

Fourteen weeks later (two weeks after completing antibiotic treatment), the wound started draining pus again. The infection was apparently not under control and we removed both plates, five cultures were taken, and extensive debridement and irrigation were performed once again. The graft appeared vital and was left in place. A Philos plate was again used as an external fixator (Figure 6). Cultures grew Streptococcus pyogenes for which he was treated with penicillin two weeks IV followed by oral clindamycin for four weeks.

**Figure 7:**
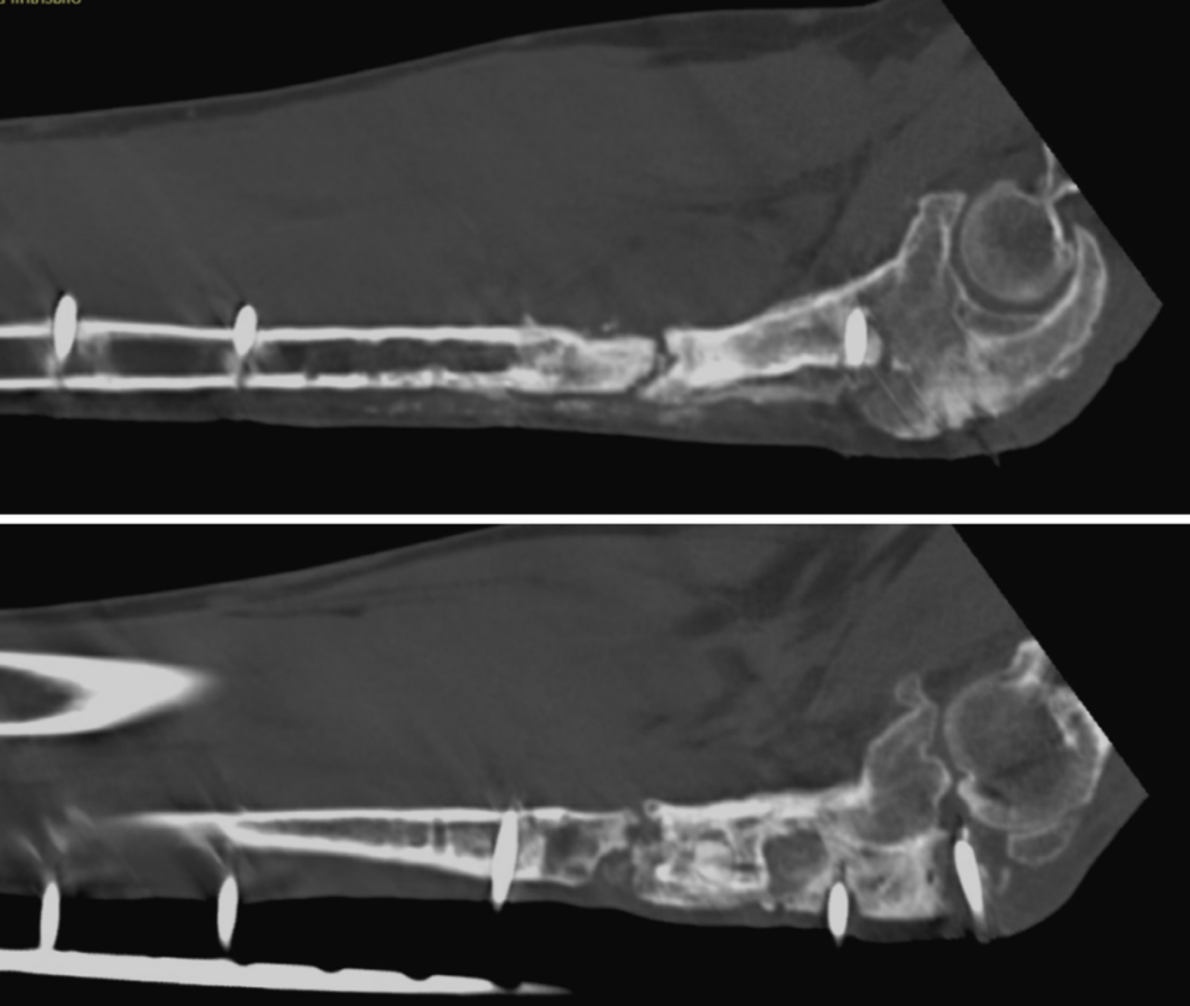
CT scan after six months The distal part of the graft is united while the proximal part is not.

At six months, there were no obvious signs of infection and function was quite good (Video 1). A CT scan showed a residual nonunion between the intercalary graft and the proximal ulna. The distal part of the nonunion was united with the intercalary graft suggesting that at least that part of the graft had incorporated successfully (Figure 7). We removed the Philos plate and placed a 3.5 titanium LCP (DePuy/Synthes, Amersfoort, The Netherlands) using a tension device to obtain maximum compression over the nonunion. The proximal junction between the graft and ulna was debrided down to bleeding bone. Additional cancellous iliac crest bone graft was added. Cultures were taken and, showed after some days, Finegoldia magna, Actinomyces neuii, Peptoniphilus harei, and Dermabacter hominis for which antibiotic treatment consisted of oral amoxicillin with oral clindamycin for 12 weeks.

**Video 1:** Six months with the plate as external fixator in situ No signs of infection and quite good function of the lower arm

The nonunion finally healed four months after our last surgery. In total, the patient underwent 12 surgeries (seven elsewhere and five in our hospital) after the initial injury six years earlier. At this point, four months following the last surgery, the patient had no pain and a good range of motion (ROM) of his elbow and wrist without infection.

**Figure 8:**
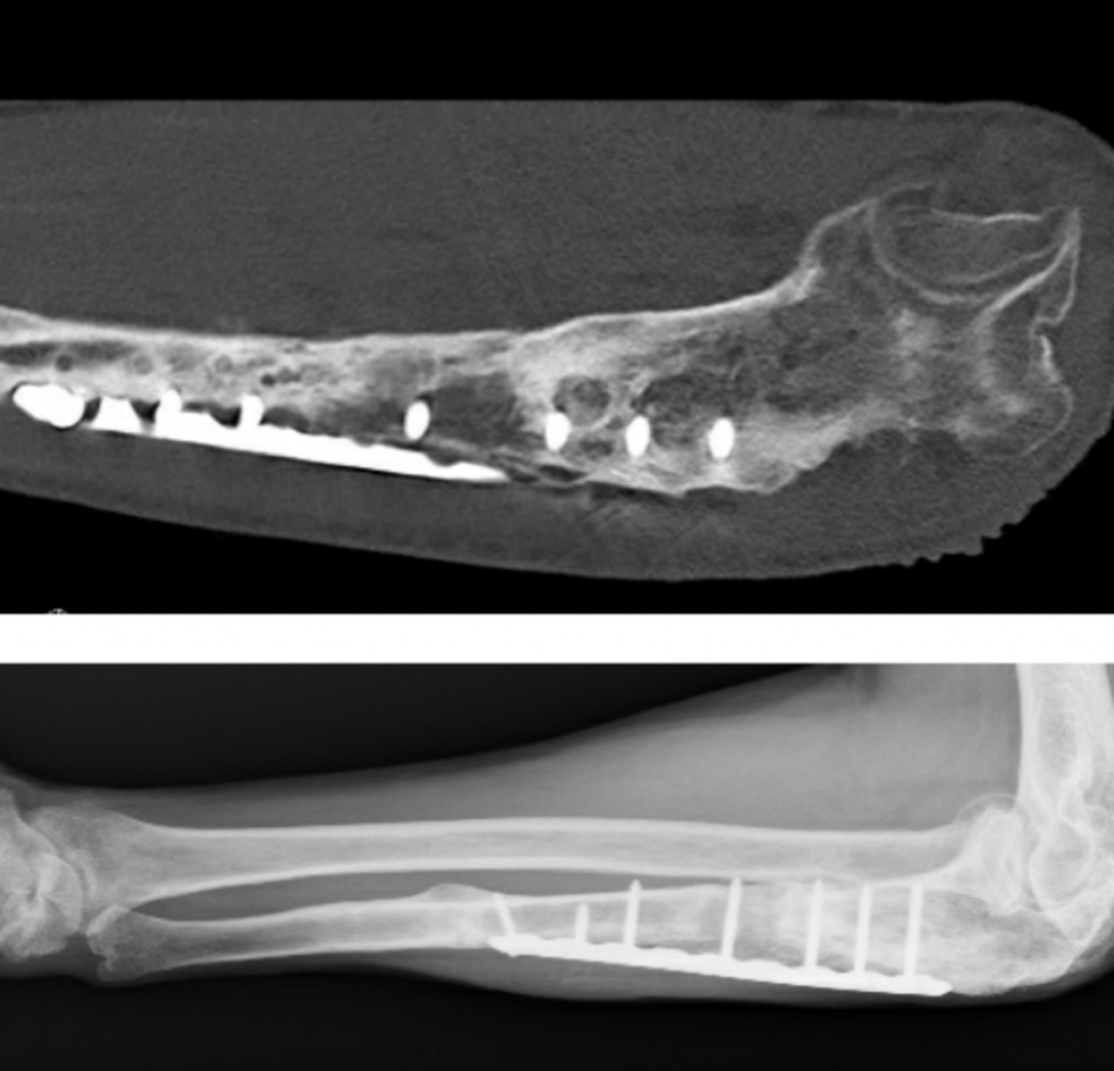
One year later and the patient presented with a fistula X-ray and CT showed complete healing and no signs of osteomyelitis.

**Figure 9:**
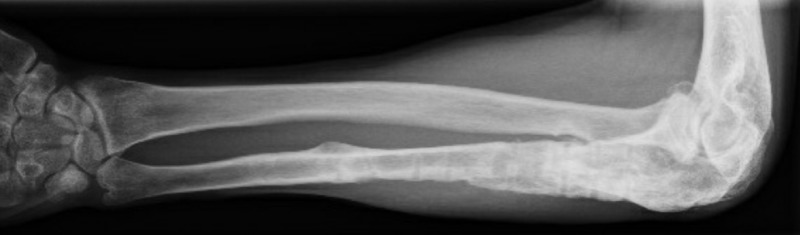
Final result after removal of the plate Showing complete healing and no signs of infection

One year later, the patient presented with a small fistula. Radiographs and CT showed complete union and no evidence of osteomyelitis or a sequester (Figure 8). The plate was removed and the skin was closed after excision of the fistula. Cultures were taken and grew Staphylococcus intermedius, Finegoldia magna, and Haemophilus parainfluenza. Antibiotic treatment consisted of oral co-cotrimoxazole and amoxicillin for six weeks. The wound healed uneventfully. Radiographs after removal of the plate showed complete healing, no shortening, and no signs of osteomyelitis (Figure 9). At the final follow-up, the patient reported a Disabilities of the Arm, Shoulder, and Hand (DASH) score of 4.2 (minor to moderate difficulty by opening a jar, changing a light bulb, or preparing a meal). He was pain-free and satisfied with a good functional result.

## Discussion

The principles of any infected (septic) nonunion treatment are debridement, antibiotics, alignment, stability, soft tissue coverage, and, most often, bone grafting [4]. When adhering to these rules, there is no reason an infected forearm nonunion cannot be healed.

Only a few reports have focused on this entity [5-9]. However, it is clear that the eradication of the infection is paramount while maintaining stability. Unfortunately, debridement will often leave a bone defect that needs to be reconstructed once the infection has been eradicated. A useful technique for this problem is the Masquelet technique that was introduced in 2000. This technique involves the placement of a PMMA spacer in the defect, which leads to the development of a membrane surrounding the cement that is rich in growth factors. Although originally not advocated by Masquelet himself, various heat-stable antibiotics, such as gentamycin, tobramycin, and/or vancomycin, can be added to the PMMA, bringing high concentrations of antibiotics in the area while maintaining space and length. At six to eight weeks, the PMMA block is removed, after which bone graft is placed in the defect. The exact mechanism of the membrane-associated osteoinduction has been the point of the ongoing discussion [10].

There have been few reports using the Masquelet technique in the upper extremity [9,11-12]. For the lower extremity, large defects up to 25 cm have been treated with the Masquelet technique using cancellous grafting. For the upper extremity, the defects filed by Masquelet have been up to 8 cm [9]. Prior to using the Masquelet technique, bone graft advocated for forearm nonunions were either autologous or homologous (cancellous or solid) or vascularized [4]. Defects of up to 6 cm can successfully be grafted with a cancellous or tricortical autograft [4]. The advantage of a solid tricortical graft is its intrinsic stability, the option to use compression plating (instead of bridging plating when using cancellous grafts), fixation of the graft itself with a screw, and rapid incorporation without the need for timely remodeling after cancellous grafting [11].

Larger defects (up to 10.5 cm) have been treated successfully with an autologous or homologous solid graft [4,8]. Defects larger than 10.5 cm need vascularized transfer [4].

Ilizarov bone transport for an (infected) forearm nonunion reconstruction has also been reported [6-7]. We think that this type of bone transport is cumbersome with a frame that is in place for many months. Overall, a vascularized bone transfer is a very powerful technique, but it requires a dedicated microsurgical team [5].

The length of antibiotic treatment depends on whether eradication or mere suppression of the infection is the goal. We discuss all our patients with a septic nonunion in a weekly conference with our microbiologists and infectious disease specialist. In this case, we were striving for eradication but clearly did not succeed in our first two attempts. In hindsight, the first set of cultures was taken under the antibiotic regime and probably were not representative. A period of five to seven days without antibiotics (antibiotic holiday) is considered to give more reliable culture results.

Stable fixation during the first stage of the Masquelet is important. Instability causes pain, necessitating immobilization with a brace or plaster, preventing easy wound control and access. It is also well-known that instability provides a poor environment for the eradication of the infection. Ideal temporary fixation during the first stage of Masquelet allows motion while leaving the smallest footprint of hardware as possible. A compromise has to be found between sufficient stability with the minimum amount of hardware possible. We were the first to describe the plate as an external fixation technique (supercutaneous plating) [13]. This technique is now used by others in the lower extremity but not so often in the upper extremity [14-15]. It is a safe, technically easy, and reproducible technique and a useful adjunct in the treatment of complex posttraumatic problems. The plate is low profile and light, allowing the patient more function than a standard external fixator does. Pin tract infections are rare with the supercutaneous plating technique [13-14].

The long-term functional outcome in our patient was compromised by residual stiffness due to the many surgeries, infection, and the long immobilization and disuse of the arm. This may have been prevented by a more structured treatment plan after the initial failure.

## Conclusions

A septic nonunion after plate fixation of a diaphyseal forearm fracture is rare. A logical, stepwise approach consisting of debridement, antibiotics, providing stability, and bone grafting is important. The points of discussion are the length of antibiotics, benefit of an antibiotic holiday, type of temporary fixation, and type of bone graft (solid or cancellous). Currently, there is no good evidence for any of these outstanding issues. Not knowing your limitations is important, as diaphyseal forearm nonunion can be a challenge. Early referral to a specialized center can prevent further complications. A multidisciplinary approach involving a radiologist, rehabilitation medicine, an infectious disease specialist, and an orthopedic trauma surgeon is needed.
